# Expression analysis of human adipose-derived stem cells during *in vitro* differentiation to an adipocyte lineage

**DOI:** 10.1186/s12920-015-0119-8

**Published:** 2015-07-24

**Authors:** Latha Satish, J. Michael Krill-Burger, Phillip H. Gallo, Shelley Des Etages, Fang Liu, Brian J. Philips, Sudheer Ravuri, Kacey G. Marra, William A. LaFramboise, Sandeep Kathju, J. Peter Rubin

**Affiliations:** Department of Plastic Surgery, University of Pittsburgh Medical Center, 3550 Terrace Street, 6B Scaife Hall, 15261 Pittsburgh, PA USA; Department of Pathology, University of Pittsburgh, Pittsburgh, PA USA; Connecticut College, Department of Biology, New London, CT USA; McGowan Institute for Regenerative Medicine, Pittsburgh, PA USA

**Keywords:** Microarray, Adipose-derived stem cells, Transcriptome, Gene expression, Stromal vascular fraction, Adipogenesis

## Abstract

**Background:**

Adipose tissue-derived stromal stem cells (ASCs) represent a promising regenerative resource for soft tissue reconstruction. Although autologous grafting of whole fat has long been practiced, a major clinical limitation of this technique is inconsistent long-term graft retention. To understand the changes in cell function during the transition of ASCs into fully mature fat cells, we compared the transcriptome profiles of cultured undifferentiated human primary ASCs under conditions leading to acquisition of a mature adipocyte phenotype.

**Methods:**

Microarray analysis was performed on total RNA extracted from separate ACS isolates of six human adult females before and after 7 days (7 days: early stage) and 21 days (21 days: late stage) of adipocyte differentiation *in vitro*. Differential gene expression profiles were determined using Partek Genomics Suite Version 6.4 for analysis of variance (ANOVA) based on time in culture. We also performed unsupervised hierarchical clustering to test for gene expression patterns among the three cell populations. Ingenuity Pathway Analysis was used to determine biologically significant networks and canonical pathways relevant to adipogenesis.

**Results:**

Cells at each stage showed remarkable intra-group consistency of expression profiles while abundant differences were detected across stages and groups. More than 14,000 transcripts were significantly altered during differentiation while ~6000 transcripts were affected between 7 days and 21 days cultures. Setting a cutoff of +/-two-fold change, 1350 transcripts were elevated while 2929 genes were significantly decreased by 7 days. Comparison of early and late stage cultures revealed increased expression of 1107 transcripts while 606 genes showed significantly reduced expression. In addition to confirming differential expression of known markers of adipogenesis (e.g., *FABP4, ADIPOQ, PLIN4*), multiple genes and signaling pathways not previously known to be involved in regulating adipogenesis were identified (e.g. *POSTN, PPP1R1A, FGF11*) as potential novel mediators of adipogenesis. Quantitative RT-PCR validated the microarray results.

**Conclusions:**

ASC maturation into an adipocyte phenotype proceeds from a gene expression program that involves thousands of genes. This is the first study to compare mRNA expression profiles during early and late stage adipogenesis using cultured human primary ASCs from multiple patients.

**Electronic supplementary material:**

The online version of this article (doi:10.1186/s12920-015-0119-8) contains supplementary material, which is available to authorized users.

## Background

Adipose-derived stem cells (ASCs) have become one of the most widely studied adult stem cell populations for soft tissue engineering and regenerative medicine applications since their isolation and characterization over a decade ago [1]. ASCs offer several advantages compared to other stem cell sources including an abundant autologous source, minimally-invasive harvesting, significant proliferative capacity in culture and multi-lineage potential. Although autologous ASC application in the United States has yet to be FDA-approved, clinical [2, 3] and pre-clinical studies [4–6] throughout the world have underscored its potential.

To date, the most significant progress for clinical application of ASCs has been in the area of whole fat grafting. However, long-term fat graft survival and durability remain unpredictable and the use of ASCs to enhance the long-term viability of fat grafts is an area of active interest. It is hypothesized that ASCs potentiate the viability of mature adipocyte grafts both as a cell source for *de novo* adipogenesis and through production of local growth factors. While animal studies demonstrate improved tissue volumes after fat grafting enriched with ASCs [4, 5], definitive studies are lacking that show that graft supplementation with ASCs is superior to standard fat grafting in humans. Understanding the molecular mechanisms that regulate the differentiation of ASCs to an adipocyte lineage can be helpful in formulating novel therapies to enhance the utility and stability of adipose tissue grafts. Limited data address temporal gene expression changes during adipogenesis. The murine 3 T3-L1 cell line is well characterized *in vitro* model for studying the conversion of preadipocytes into adipocytes since derivation in the early 1960’s by Howard Green [7]. Several studies [8–12] have utilized this cell line for examination of gene expression changes during adipocyte development as these cells readily accumulate lipid upon differentiation. In addition, murine [13] and porcine [14] adipose tissues have been examined for transcriptional profile comparisons with human adipose tissue. However, with the recent discovery of human ASCs [1, 15], these multi-potent stem cells have become the main focus for analyzing transcriptional and protein profile changes during differentiation [16–19].

To better understand the cellular and physiologic processes during the transition of ASCs toward the adipocyte phenotype, we characterized and compared the transcriptome profiles of undifferentiated human primary ASCs prior to and after undergoing adipocyte differentiation for 7 (early stage) and 21 days (late stage) under conditions inducing adipogenic differentiation.

## Methods

### Adipose tissue collection

Subcutaneous adipose tissue was harvested during elective abdominoplasties from six human adult female patients. Mean age of the patients was 43.7 (±10.3) years and mean BMI was 37.8 (±15.1). All subjects were in good health and free from diabetes. These studies were carried out under an exempt protocol with no consent required from the subjects as it is discarded tissue used without any personal identifying information. Protocol was approved by the University of Pittsburgh’s Institutional Review Board under the IRB protocol # PRO13090506. These studies have been conducted according to the principles expressed in the Declaration of Helsinki.

### Human adipose-derived stem cell (ASC) isolation

Adipose-derived stem cells (ASCs) were isolated from abdominal subcutaneous adipose tissue as previously described [1] with minor modifications. Briefly, adipose tissue was first minced with large surgical scissors and digested in Hank’s Balanced Salt Solution (Invitrogen, Carlsbad, CA) containing 3.5 % bovine serum albumin (Millipore, Charlottesville, VA) and 1 mg/ml collagenase type-II (Worthington Biochemical Corp., Lakewood, NJ). The tissue was then gently shaken in a 37 °C water bath for 40 min and centrifuged at 180×*g* for 10 min. The resulting cellular pellet (= stromal vascular fraction, SVF) was then resuspended in ammonium chloride-based erythrocyte lysis buffer (Beckman Coulter, Miami, FL), and centrifuged at 180×*g* for 10 min. Next, the SVF pellet was resuspended in 20 ml ASC plating media (pH 7.4, DMEM:DMEM/F12 (1:1), 10 % fetal bovine serum (FBS), 1 % penicillin/streptomycin, Invitrogen; 0.5 % Fungizone, Fisher Scientific, Pittsburgh, PA; 0.001 % dexamethasone, Sigma-Aldrich, St. Louis, MO), disaggregated through sterile cotton gauze (12-ply, Fisher Scientific), then plated on tissue culture-treated flasks (BD Biosciences, Franklin Lakes, NJ). After overnight incubation, non-adherent cells were removed by gentle aspiration and fresh ASC plating medium was added to the flasks. ASCs were maintained until near confluence (passage 0: 24–48 h), and then harvested and stored in liquid N_2_ until use.

### Adipogenic differentiation

Asynchronous ASCs (passage 1) from each patient were seeded on tissue culture treated flasks (BD Biosciences) at approximately 80 % confluence and cultured with ASC plating media until nearly 100 % confluence. Plating media was then gently aspirated and replaced with adipocyte differentiation medium (Zen-Bio Inc., Research Triangle Park, NC). The differentiation media was replaced every other day for either 7 or 21 days. The cells were maintained in the differentiation medium throughout the entire time period of differentiation. Cells cultured in ASC plating media served as undifferentiated Day 0 controls. At the end of each time period, ASC differentiation (to mature adipocytes) was quantified by intracellular lipid accumulation using the AdipoRed Assay Reagent as described below. The differentiation media was replaced every other day for 21 days.

### Adipored assay

Undifferentiated ASCs were plated at a density of 5 × 10^5^ cells/35 mm dish and at 1 × 10^3^ cells/well in 96-well plates and allowed to differentiate for a period of 7 and 21 days. Cells cultured in ASC plating media served as Day 0 controls. On days 0, 7 and 21, cells were treated with AdipoRed (Lonza Inc., Allendale, NJ), and total lipid content was determined according to the manufacturer’s protocol. In brief, cells grown in 35 mm dishes were washed twice with phosphate-buffered saline (PBS), followed by the addition of 2 ml of PBS containing 60 μl of AdipoRed reagent and incubated for 15 min at room temperature. AdipoRed becomes fluorescent when partitioned in a hydrophobic compartment. Phase contrast and fluorescence images were captured using an inverted Nikon Eclipse TE2000-U fluorescent microscope (Nikon Inc. Melville, NY). Cells grown in 96 well plates were washed twice with PBS followed by the addition of 200 μl of PBS containing 5 μl of AdipoRed reagent and incubated in room temperature for 15 min. The fluorescence of each well was measured using a Tecan Infinite M200 PRO (Tecan US, Morrisville, NC) at 485 nm excitation and 572 nm emission wavelengths.

### RNA extraction

Total RNA from undifferentiated ASCs, and cells subjected to 7 and 21-day differentiated protocols was extracted using the RNeasy Mini Kit (Qiagen Inc., Valencia, CA) according to the manufacturer's instructions. The concentration of the extracted RNA was quantified using a Nanodrop ND-1000 Spectrophotometer (NanoDrop, Wilmington, DE) and only samples with absorption ratio 260/280 ≥ 1.8 were subjected to further processing. The purified RNA samples were characterized for size and integrity using the Agilent 2100 Bioanalyzer (Agilent Technologies, Santa Clara, CA) to ensure sample quality and absence of degradation (RIN value > 8.0).

### Microarray

Total RNA purified from undifferentiated ASCs (*n* = 5), as well as cells subjected to differentiation conditions for 7 days (*n* = 4) and 21 days (*n* = 6) were subjected to array analysis. *In vitro* transcription was performed using the Ambion Message Amp Premier Enhanced assay protocol (Ambion Inc, Austin, TX) starting with 500 ng of purified total RNA. Confirmation of cRNA diversity was obtained using the Bioanalyzer 2100 to characterize sample yield, integrity, and size diversity against a Universal Human Reference RNA (Stratagene, La Jolla, CA). Fifteen micrograms of purified, amplified, biotin labeled cRNA was fragmented and hybridized onto Affymetrix Human Genome HGU133A plus 2.0 arrays (Affymetrix Corp., Santa Clara, CA) for 18 h. Washing, staining and scanning of arrays was performed on the Affymetrix Fluidics Station 450 and Scanner 3000 immediately after completion of hybridization. The preparation of cRNA and hybridization to microarrays was performed by Gene Logic Inc. Data quality was assessed by using pre-normalized data to generate degradation plots and compare 3′–5′ rations for all the actin and GAPDH probesets [20]. Microarray data was processed using the Expression Console (Affymetrix) with signal intensity calculated by Microarray Suite version 5.0 (MAS 5.0).

### Microarray data analysis

Data analysis to determine differential gene expression profiles was performed by importing MAS 5.0 intensity data into the Partek Genomics Suite Version 6.4 (St. Louis, MO) for analysis of variance (ANOVA) based on time in culture. Post hoc testing employed the Fisher’s exact test adjusted to correct for false discovery rates (*q* < 0.05) associated with multiple testing. We also performed unsupervised hierarchical clustering to test for gene expression patterns among the three cell populations. In order to determine biologically significant networks and canonical pathways relevant to adipogenesis, we utilized Ingenuity Pathway Analysis (IPA Suite, Ingenuity Systems; http://www.ingenuity.com, Mountain View, CA). Only differentially expressed genes (−2.0 > fold-change >2.0; *q* value <0.05) were included in each IPA core analysis. Genes were mapped to genetic networks and statistically ranked based on the number of differentially expressed genes among the eligible molecules in the network. Microarray data sets obtained through this study will be deposited in NCBI’s Gene Expression Omnibus (GEO).

### Quantitative reverse transcription- PCR

The protocols for reverse transcription reactions and real-time PCR were previously described [21]. Pooled, total RNA (400 ng) from each of the three cell populations (undifferentiated ASCs, 7- and 21-day differentiated ASCs) were mixed with 300 ng of random primers (Invitrogen Corporation) and reverse transcribed in 60 μl reactions. Real-time PCR amplification and detection of templates were performed in triplicate assays carried out on an Applied Biosystems PRISM 7900HT system using Applied Biosystems Taqman transcript-specific assays as listed in Table 1. Using the comparative critical cycle (Ct) method and using glyceraldehyde phosphate dehydrogenase (GAPDH) as the endogenous control, the expression levels of the target genes were normalized using a 95 % confidence interval. The relative expression of the genes from each sample set was averaged and statistical analysis for significance was performed using a Student’s t-test.

**Table 1:** Taqman Probes used for QRT-PCR Assay

PROBES	TAQMAN SPECIFIC TRANSCRIPT ASSAY ACCESSION NO
Adiponectin	Hs00605917_m1
Fatty acid binding protein (FABP4)	Hs01086177_m1
Fibroblast growth factor-1 (FGF1)	Hs00265254_m1
Fibroblasts growth factor-11 (FGF11)	Hs00182803_m1
Hairy and enhancer of split-1 (Hes1)	Hs00172878_m1
Perilipin 4 (PRLN4)	Hs00287411_m1
Periostin (POSTN)	Hs00170815_m1
Protein phosphatase 1, regulatory (inhibitor) subunit 1A (PPP1R1A)	Hs00410058_m1
Radical S-adenosyl methionine domain containing 2 (RSAD2)	Hs00369813_m1
GAPDH	Hs02758991_g1

## Results

### Adipored confirmation of differentiation to adipocyte lineage

**Fig. 1:**
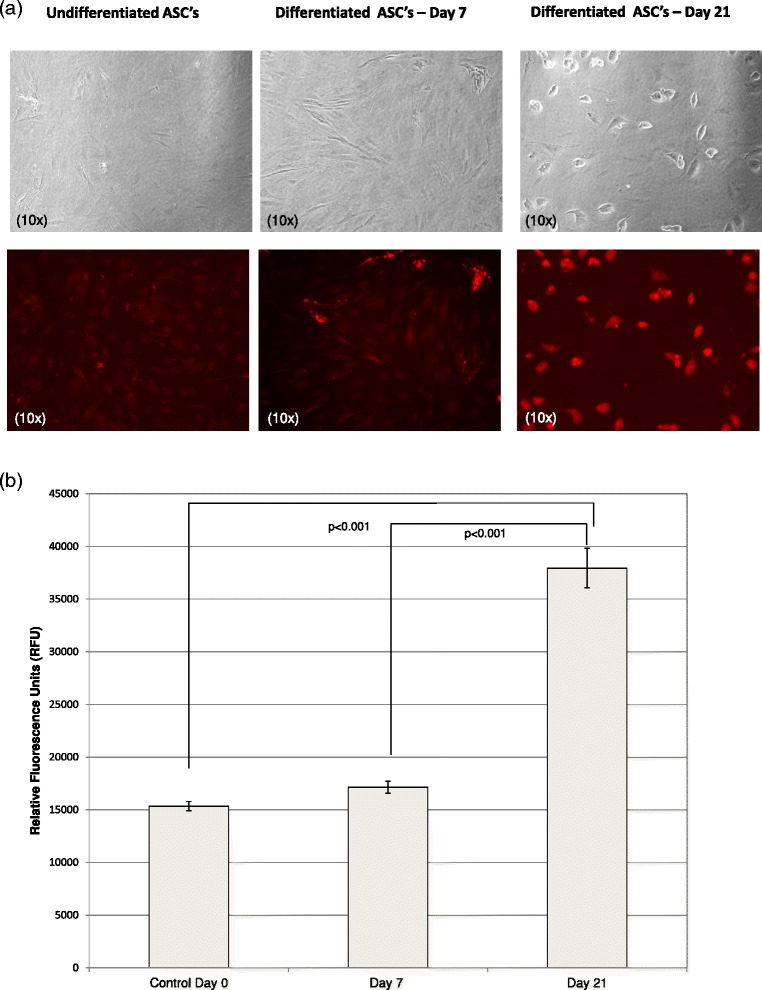
Intracellular lipid accumulation in undifferentiated, 7- and 21-day differentiated ASCs: **a** Phase contrast images of cells (10x magnification) are presented in the top panel and the corresponding AdipoRed stained images are shown in the bottom panel. These are representative photographs from two independent experiments each performed in duplicate. **b** Quantification of intracellular lipid showed significantly increased accumulation of lipid droplets in 21-day differentiated ASCs versus undifferentiated and 7-day differentiated ASCs. Values represent mean ± SEM of two independent experiments performed in triplicate. Statistical analysis was performed using Student’s *t* test and *p* < 0.05 was considered significant

The AdipoRed assay was employed to monitor accumulation of intracellular lipid at 7 and 21 days of adipogenic cell culture media consistent with a protocol directing differentiation into the adipocyte phenotype. Under the microscope 21-day differentiated cells showed increased fine red-colored lipid droplets bounded by the cell membrane compared to both undifferentiated and 7-day differentiated ASCs (Fig. 1a). Quantitative analysis of intracellular lipid accumulation based on cellular fluorescence confirmed the increased levels of lipid present with time in differentiation media (Fig. 1b).

### Microarray analysis of differential gene expression

Comprehensive RNA degradation plots of individual mean probe intensities indicated comparable transcript integrity across all samples and culture periods (Additional file 1a). Box plot analysis of individual array sample intensities demonstrated comparable dispersion of the individual data sets, regardless of patient source, eliminating the need for additional normalization based on constitutive markers and secondary smoothing of the raw intensity data prior to statistical evaluation (Additional file 1b). Furthermore, these results also indicated that each sample set exhibited a comparable broad diversity of transcripts unaffected by noise and not skewed by outliers.

**Fig. 2:**
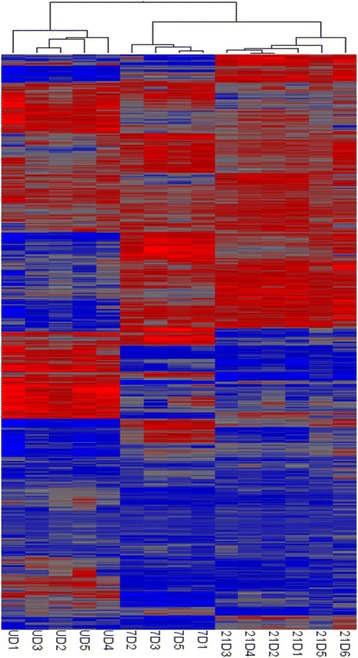
Hierarchical clustering and heat map analysis of individual gene expression profiles: A heat map depiction of the transcriptomic profiles of all samples analyzed by microarray is presented. Red bars indicate relatively high signal intensity for the gene product in question, with blue representing lower intensity and grey intermediate. Clustering of the samples is indicated by the dendrogram on top; it is apparent that all 5 undifferentiated samples cluster together (have the highest degree of similarity with each other), and the same is true for the 4 7-day differentiated samples and the 6 21-day differentiated samples. UD 1–5: undifferentiated ASCs; 7days 1–3, 5: 7-day differentiated ASCs; 21 day 1–6: 21-day differentiated ASCs

Gene expression in 0 day control (undifferentiated) cells differed significantly from those harvested after 7 and 21 days of differentiation (ANOVA: *p* < 0.02). Post-hoc testing revealed that approximately 14,000 transcripts were significantly altered between control and either day 7 or day 21 cultures. Comparison of expression values between day 7 and day 21 revealed that approximately 6,000 different transcripts were significantly altered between these two time points after correction for false discovery (*q* < 0.05). In comparing expression between day 7 and day 0, 1350 genes exhibited > two-fold increase while 2929 genes fell more than two-fold. Comparing 21 day vs 7 day differentiated cells, expression levels of 1107 transcripts increased and 606 genes decreased (Table 2 and Additional files 2 and 3). These data indicate that ASC differentiation *in vitro* is a dynamic and complex process affecting a dominant portion of the transcriptome. Unsupervised hierarchical clustering reinforced these findings with each cell classification forming discriminant clusters (Fig. 2). There were notable differences in the expression of some transcriptional regulators involved in maintenance of stem cell pluripotency on Day 7 and Day 21 vs undifferentiated controls and the shift from a stem cell phenotype to a mature adipocyte was accompanied by a significant reduction in NANOG expression (data not shown).

**Table 2:** Identification of Differentially Regulated Genes Comparing 7 day vs Undifferentiated cells and 21 day vs 7 day cells

Days	Upregulated Genes (2 fold and above)	Downregulated Genes (0.5 fold and below)
7 day vs Undiff	1350	2929
21 day vs 7 day	1107	606

### Quantitative RT-PCR analysis of selected transcripts

**Fig. 3:**
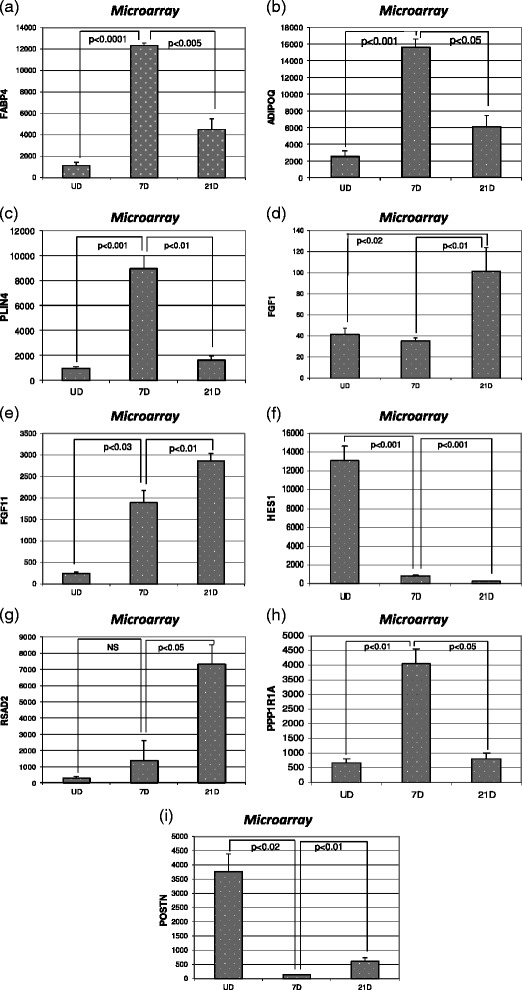
Relative expression levels of select genes as determined by microarray: Histogram presentations of the relative expression levels of (**a**) FABP4 (**b**) ADIPOQ (**c**) PLIN4 (**d**) FGF1 (**e**) FGF11 (**f**) HES1 (**g**) RSAD2 (**h**) PPP1R1A (**i**) POSTN as quantified by microarray are shown. Signal intensities for each gene product are expressed in arbitrary units after background subtraction. Statistical significance was determined by Student’s *t* test and *p* value < 0.05 was considered significant

**Fig. 4:**
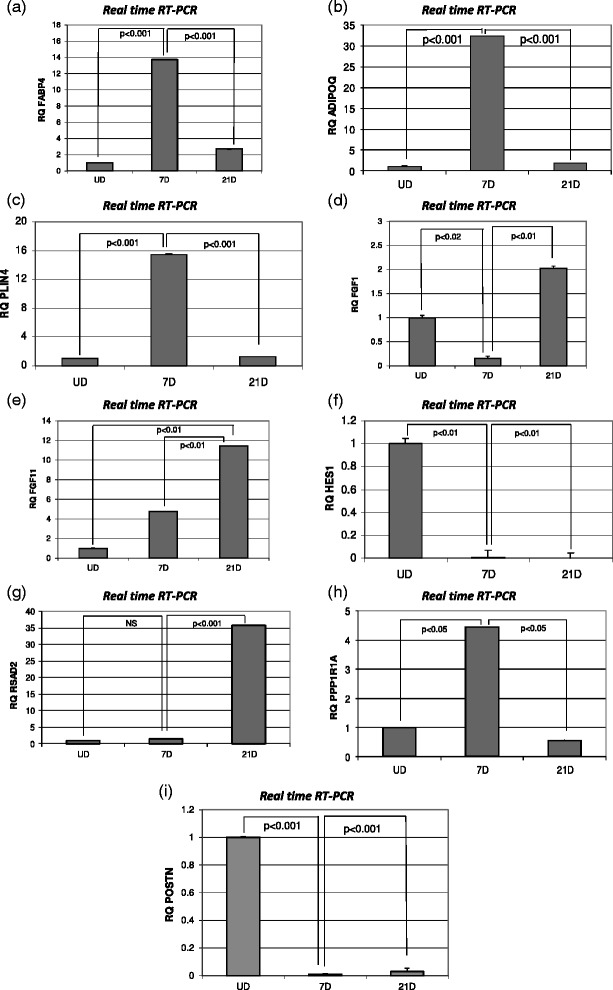
Confirmation of select gene expression by direct quantitative RT-PCR assay: The results of direct quantitative RT-PCR assays measuring relative message levels of (**a**) FABP4 (**b**) ADIPOQ (**c**) PLIN4 (**d**) FGF1 (**e**) FGF11 (**f**) HES1 (**g**) RSAD2 (**h**) PPP1R1A (**i**) POSTN are shown as histograms. Relative values for each transcript were normalized by internal control (GAPDH), and are shown here in each instance with baseline expression in undifferentiated ASCs set at a relative value of “1”. Values represent mean ± SEM of two independent experiments performed in triplicate. *p* < 0.05 was considered significant. In all cases a strong concordance was seen in the patterns of gene expression measured by microarray and measured by direct qRT-PCR

We performed direct quantitation of nine selected gene products by real-time RT-PCR to validate the microarray findings. Genes were selected based on their established relevance to adipocyte biology, or were included to validate their heretofore unknown importance to adipocyte maturation. In every case, a strong correspondence between the microarray and real time RT-PCR data was observed (Figs. 3 and 4).

Expression levels of FABP4 (fatty acid binding protein 4 or adipocyte protein 2), ADIPOQ (adiponectin) and PLIN4 (perilipin), known markers for differentiated adipocytes, were significantly increased at 7 and 21 days in culture albeit with a statistically significant decline after day 7 (Figs. 3a,b,c and 4a,b,c). Similarly, PPP1R1A (protein phosphatase 1, regulatory (inhibitor) subunit 1A), was markedly elevated at 7 days versus undifferentiated controls, but fell back to baseline by 21 days (Figs. 3h and 4h). We also investigated fibroblast growth factors identified as differentially expressed by the microarray. Fibroblast growth factor 1 (FGF1) was initially decreased in 7days culture but showed a significant increase well above the 0day baseline at 21 days in culture. In contrast, fibroblast growth factor 11 (FGF11) showed both an initial increase at 7 days, and a further increase at 21 days (Figs. 3d,e & 4d,e).

Two genes, periostin (POSTN) and HES1 (hairy and enhancer of split-1), exhibited decreased expression in both 7days and 21day cultures relative to undifferentiated cultures: (Figs. 3f4f3i & 4i). Finally, RSAD2 (radical S-adenosyl methionine domain containing 2) showed no significant change after 7 days of culture but was dramatically elevated at 21 days (Figs. 3g & 4g).

### Identification of significant biological pathways using ingenuity analysis

To investigate the biological function of differentially modulated genes, transcripts reflecting statistically significant alterations (*q* < 0.05, −2 > fold-change > +2) were interrogated by Ingenuity Pathway Analysis (IPA). In comparing 7days vs undifferentiated cells, and 21day vs 7days cultures, the top five canonical pathways, top networks, top molecules, and top transcription factors identified are listed in Tables 3a–d and 4a–d respectively. For 7 day versus undifferentiated ASCs, most of the top canonical pathways and networks identified in this study have been previously shown to play a role in adipogenesis, with four of the five networks explicitly involved in lipid metabolism. However, examination of the top molecules for the first time identifies CXCL6, a CXC chemokine previously shown to have angiogenic properties, as a potential regulator of adipogenesis, and also identifies thrombospondin as potentially important (Table 3c). Analysis of data on d21 vs d7 was interesting in that the most prominent pathway, TREM-1 (triggering receptor expressed on myeloid cells 1) signaling, is known to activate a cascade of events associated with inflammatory processes. Top molecules identified in this comparison comprised interferon-induced genes such as RSAD2, HECT and RLD domain containing E3 ubiquitin protein ligase family member 6 (HERC6), along with the chemokine CCL5 and tumor necrosis factor superfamily member 15 (TNFSF15) (Table 4c). Transcription factor analysis predicted that PPAR and SREB transcription factors, key regulators of adipocyte physiology, played prominent roles in 7days vs undifferentiated cultures (Table 3d), but did not have a dominant role in the transition from 7days to 21day (Table 4d).

**Table 3:** Top Canonical Pathways (a) and Top Networks (b) Identified Using Ingenuity Pathway Analysis in Comparing 7 day vs Undifferentiated Cells: Top Molecules (c) and Top Transcription Factors (d) Identified Using Ingenuity Pathway Analysis in Comparing 7 day vs Undifferentiated Cells

(a)		(b)		(c)		(d)		
Name	*p*-value	Associated Network Functions	Score	Name	Exp.Value	Transcription Regulator	*P*-value of overlap	Predicted Activation State
LPS/IL-1 Mediated Inhibition of RXR Function	6.17E-08	Lipid Metabolism, Small Molecule Biochemistry, Gastrointestinal Disease	39	PCK1	−40.310	PPARG	2.59E-22	Activated
Pyruvate Metabolism	6.28E-08	Drug Metabolism, Protein Synthesis, Cell Death	39	THRSP	−31.613	PPARA	1.02E-20	Activated
Fatty Acid Biosynthesis	3.49E-07	Lipid Metabolism, Molecular Transport, Small Molecule Biochemistry	36	CA3	−28.877	SREBF1	5.80E-18	-
TR/RXR Activation	1.65E-06	Lipid Metabolism, Small Molecule Biochemistry, Energy Production	35	BBOX1	−28.336	SREBF2	1.60E-10	-
Glycolysis/Gluconeogenesis	1.34E-05	Lipid Metabolism, Molecular Transport, Small Molecule Biochemistry	35	GPD1	−26.297	PPARGC1A	3.23E-10	-
				PDE3B	−19.778			
				SCD	−17.679			
				CXCL6	−17.037			
				LGALS12	−16.318			
				FABP4	−14.682

**Table 4:** Top Canonical Pathways (a) and Top Networks (b) Identified Using Ingenuity Pathway Analysis in Comparing 21 day vs 7 day

(a)		(b)			(c)		(d)		
Name	*p*-value	ID	Associated Network Functions	Score	Name	Exp.Value	Transcription Regulator	*P*-value of overlap	Predicted Activation State
TREM1 Signaling	1.49E-08	1	Dermatological Diseases and Conditions, Genetic Disorder, Organismal Injury and Abnormalities	43	RSAD2	−33.001	IRF7	5.14E-37	-
Communication between Innate and Adaptive Immune Cells	2.27E-08	2	Cellular Response to Therapeutics, Cellular Assembly and Organization, Cellular Compromise	33	CMPK2	−16.965	STAT3	2.26E-27	-
Hepatic Fibrosis/Hepatic Stellate Cell Activation	6.52E-08	3	Inflammatory Disease, Neurological Disease, Cellular Function and Maintenance	31	ORM1/ORM2	−16.674	NFkB (complex)	2.48E-26	-
Interferon Signaling	8.34E-08	4	Lipid Metabolism, Small Molecule Biochemistry, Molecular Transport	31	CCL5	−15.185	IRF1	1.73E-22	-
Role of Pattern Recognition Receptors in Recognition of Bacteria and Viruses	2E-07	5	Cellular Assembly and Organization, Cellular Function and Maintenance, Cellular Movement	31	LOC152742	−13.169	IRF3	5.54E-22	-
					HERC6	−11.919			
					MX1	−11.250			
					OAS3	−11.105			
					IFI27	−10.472			
					TNFSF15	−10.235			

## Discussion

Human primary adipose-derived stem cells exhibited vast changes in mRNA expression profiles throughout transformation (~14,000 transcripts) from undifferentiated, stem cells (0 day) to late stage (21 day) lipid-laden, adipocytes of mature phenotype. Despite these dynamic transitions, cells in each stage demonstrated consistent intra-group transcriptome profiles and distinct expression pattern clusters. It is important to consider that the original stromal vascular fraction (SVF) from which our ASC cultures were derived comprised a mix of at least four different constituent cell types, and transcript levels represented summated expression of these cell sub-populations. However, the most prevalent and proliferative cell type within the SVF is the pre-adipocyte cell, typically characterized as CD31(−)/CD34(+), and this unique primary cell culture model likely reflects the dominant influence of those ASCs [22].

Our data confirm the importance of several key adipocyte-specific genes known to be downstream targets of PPARG including FABP4, ADIPOQ, and PLIN4. These transcripts dramatically increased in expression after 7 days but returned toward baseline by 21 days, suggesting a critical role for these genes during induction of the adipocyte differentiation program but diminution of importance in maintenance of the mature phenotype. A similar pattern of expression was seen in PPP1R1A transcripts, the inhibitory regulatory subunit 1A of protein phosphatase 1. This gene is important in glycogen metabolism [23], and has been proposed as a marker for pancreatic β-cell injury [24]. Elevated PPP1R1A expression at 7 days suggests that inhibition of protein phosphatase activity contributes to early stages of differentiation in ASCs and supports the possibility that chemical inhibitors of protein phosphatase 1 may drive ASCs toward a mature phenotype.

We also characterized the differential expression of fibroblast growth factor 1 (FGF1) and FGF11 during adipogenesis. Several FGF family members have previously been identified as potent adipogenic factors secreted from adipose-derived microvascular endothelial cells (MVECs). FGF1 stimulates all stages of adipogenesis including preadipocyte proliferation, commitment and differentiation [25]. A role for FGF1 in adipose is also supported by the demonstration of defects in vasculature and adipocyte size in the fat depots of FDF1 deficient mice [26]. Its low levels after 7 days of culture followed by a distinct increase at 21 days suggests that this factor plays a critical role in establishing and maintaining the mature adipocyte phenotype. FGF11 expression increased at the 7 day time point and rose further by 21 days. This is the first report of changes in FGF11 associated with adipogenesis. Elevated FGF11 expression has been noted in neural precursor cells [27] and cardiomyocytes [28] and increased FGF11 has also been associated with lymphatic spread of an oral squamous cell carcinoma cell line [29].

Another gene product showing dramatically increased late stage expression is RSAD2, (radical S-adenosyl methionine domain-containing protein 2) commonly known as viperin. Viperin is an interferon-inducible gene active against a range of viral pathogens, including dengue, West Nile, human immunodeficiency virus (HIV), and cytomegalovirus (CMV). No role for viperin in adipogenesis has previously been described, but viperin protein can disrupt lipid rafts increasing lateral mobility of the plasma membrane [30], and is known to localize to lipid droplets in cells. Its anti-viral function in hepatitis C virus is thought to be associated with the ability to ameliorate viral replication in lipid droplets [31]. RSAD2 is also a candidate gene that maps to a chromosome 12 quantitative trait locus associated with adipose composition [32]. Our 21 day differentiated adipocytes are rich depositories of accumulated lipids and it may be that viperin is concomitantly synthesized and accumulated in concert with this process which needs to be validated.

We identified a marked inhibition of HES1 and periostin expression during the adipocyte differentiation process. HES1 is a powerful basic helix-loop-helix transcription factor associated with regulation of tissue specificity during development. Reduction in HES1 expression during differentiation suggests this factor is critical to maintenance of the committed but undifferentiated adipocyte stem cell. These data are consistent with previous reports that HES1 expression decreases during adipocyte differentiation in the 3 T3-L1 system *in vitro* and *in vivo* [12] while constitutive over-expression inhibits differentiation of 3 T3-L1 preadipocytes. Periostin is an extracellular integrin ligand expressed at high levels in bone and teeth but involved in a host of cellular and disease processes [33]. Periostin has been implicated in cardiac development and pathophysiology [34], tumor survival and invasion [35], and the fibroproliferative disorder Dupuytren’s contracture [36]. A role for periostin in adipogenesis has not previously been reported. Exploring the role of HES1 and periostin in adipocyte biology might provide more insights in modulating the functions of adipocytes.

The magnitude of changes across the transcriptome of stem cells undergoing differentiation into mature adipocytes in this study was surprising. We were able to confirm the role of several genes previously associated with this process while revealing a number of new transcripts directly affected. Substantial work remains to classify the underlying regulatory mechanisms driving the process. However, the reproducibility of our results across multiple primary cell isolates at each stage indicates that this primary cell culture model has the necessary fidelity for demanding mechanistic studies. The ultimate physiological and clinical significance of these findings will likely arise from molecular and pharmacological manipulation of targets and pathways identified in this model.

## Conclusions

This study for the first time determines differential mRNA expression profiles during early- and late- adipocyte differentiation from ASC precursors and identifies multiple new transcripts which may be potential targets for enhancing the retention of fat grafts.

## Additional file

Additional file 1:
**a: RNA degradation profiles for generated cRNA probes: RNA degradation plots for probes generated from each individual sample are presented.** Mean intensity of the probe was plotted as a function of 5’–3’ position. All probes demonstrated a comparable slope, indicating similar integrity across sample probes. b: Box plot analysis comparing microarray signal intensities from undifferentiated, 7- and 21-day differentiated ASCs: Box plot evaluation of signal from each hybridized sample showed a comparable broad diversity of hybridized transcripts unaffected by noise and not skewed by outliers, eliminating the need for normalization or smoothing of the raw data. (a) 7-day vs undifferentiated ASCs; (b) 21-day vs 7-day ASCs. (ZIP 206 kb) Additional file 2:
**Excel sheet showing differential gene expression comparing Day 7 and Day 0 samples. **(XLSX 345 kb) Additional file 3:
**Excel sheet showing differential gene expression comparing Day 21 and Day 7 samples.** (XLSX 132 kb)
