# Supplementary figures and images for: Expression analysis of human adipose-derived stem cells during in vitro differentiation to an adipocyte lineage
Source: BMC Med Genomics. 2015 Jul 24;8:41. doi: 10.1186/s12920-015-0119-8 (PMC4513754; doi:10.1186/s12920-015-0119-8)

**Supplemental File 1a**

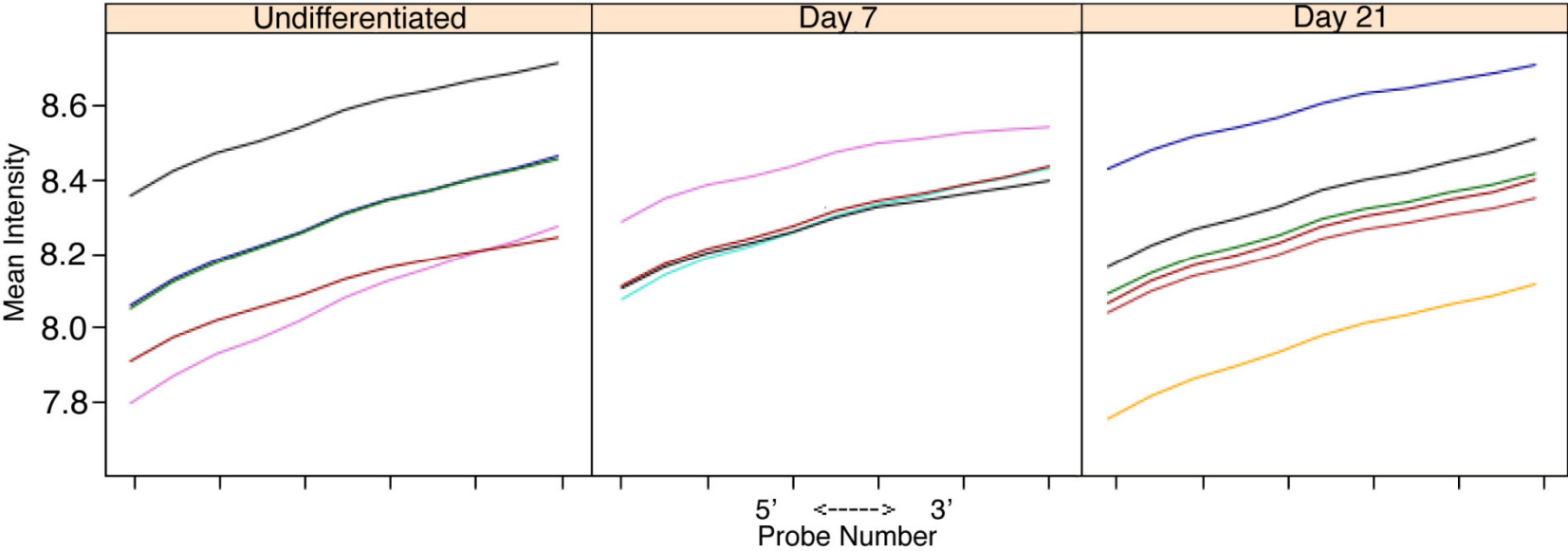

Supplement: Additional file 1: — a: RNA degradation profiles for generated cRNA probes: RNA degradation plots for probes generated from each individual sample are presented. Mean intensity of the probe was plotted as a function of 5’–3’ position. All probes demonstrated a comparable slope, indicating similar integrity across sample probes. b: Box plot analysis comparing microarray signal intensities from undifferentiated, 7- and 21-day differentiated ASCs: Box plot evaluation of signal from each hybridized sample showed a comparable broad diversity of hybridized transcripts unaffected by noise and not skewed by outliers, eliminating the need for normalization or smoothing of the raw data. (a) 7-day vs undifferentiated ASCs; (b) 21-day vs 7-day ASCs. (ZIP 206 kb) [file 12920_2015_119_MOESM1_ESM.zip]

## Supplemental File 1b

(a)

**Box & Whisker Plots (7D vs UD)**

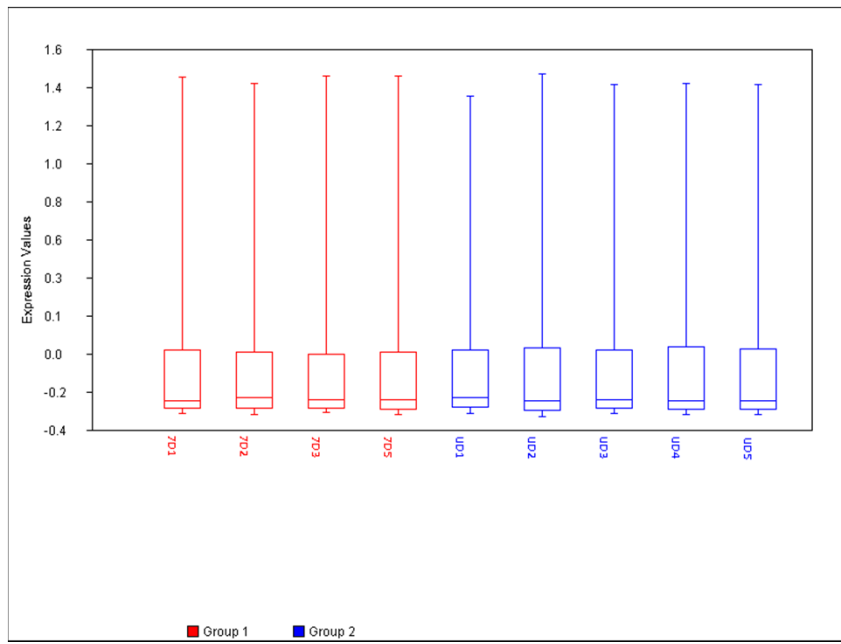

(b)

**Box & Whisker Plots (21D vs 7D)**

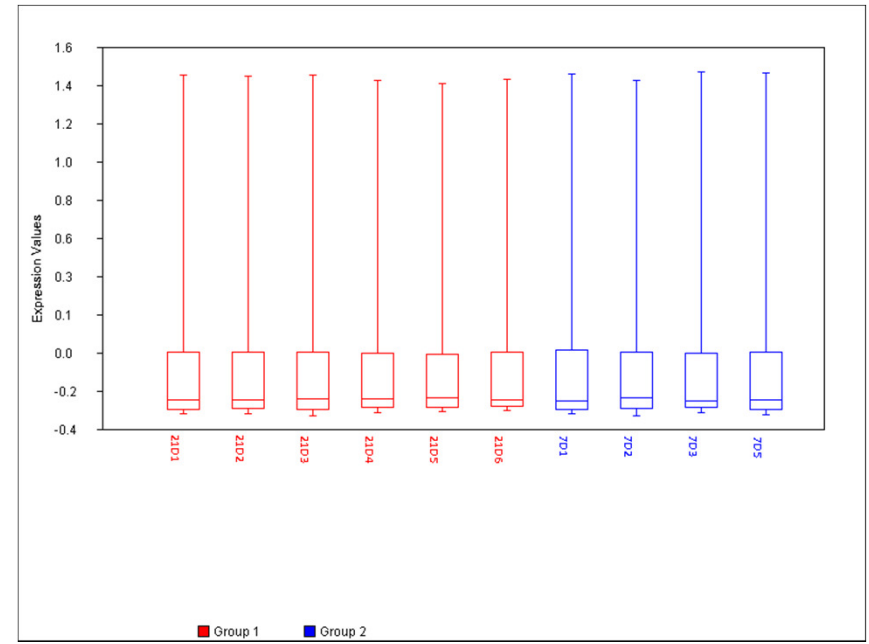

Supplement: Additional file 2: — Excel sheet showing differential gene expression comparing Day 7 and Day 0 samples. (XLSX 345 kb) [file 12920_2015_119_MOESM2_ESM.xlsx]
